# Paths and determinants for *Penicillium janthinellum* to resist low and high copper

**DOI:** 10.1038/srep10590

**Published:** 2015-08-12

**Authors:** Jian Xu, Guo-Li Chen, Xue-Zhe Sun, Xian-Wei Fan, Li You-Zhi

**Affiliations:** 1 State Key Laboratory for Conservation and Utilization of Subtropical Agro-bioresources; Key Laboratory of Ministry of Education for Microbial and Plant Genetic Engineering; College of Life Science and Technology, Guangxi University; 100 Daxue Road, Nanning, Guangxi 530004, P. R. China

## Abstract

Copper (Cu) tolerance was well understood in fungi yeasts but not in filamentous fungi. Filamentous fungi are eukaryotes but unlike eukaryotic fungi yeasts, which are a collection of various fungi that are maybe classified into different taxa but all characterized by growth as filamentous hyphae cells and with a complex morphology. The current knowledge of Cu resistance of filamentous fungi is still fragmental and therefore needs to be bridged. In this study, we characterized Cu resistance of *Penicillium janthinellum* strain GXCR and its Cu-resistance-decreasing mutants (EC-6 and UC-8), and conducted sequencing of a total of 6 transcriptomes from wild-type GXCR and mutant EC-6 grown under control and external Cu. Taken all the results together, Cu effects on the basal metabolism were directed to solute transport by two superfamilies of solute carrier and major facilitator, the buffering free CoA and Acyl-CoA pool in the peroxisome, F-type H^+^-transporting ATPases-based ATP production, V-type H^+^-transporting ATPases-based transmembrane transport, protein degradation, and alternative splicing of pre-mRNAs. Roles of enzymatic and non-enzymatic antioxidants in resistance to low and high Cu were defined. The backbone paths, signaling systems, and determinants that involve resistance of filamentous fungi to high Cu were determined, discussed and outlined in a model.

Copper (Cu) is known as one of the trace nutrient metals, which is essential at low concentrations but toxic at high concentrations^1^,^2^. The mechanisms of Cu resistance have been proposed in bacteria^3^, yeasts^4^, mammalian^5^, *Drosophila*^6^, plants^7^, and crustaceans^8^, showing great differences with species. For example, plasmid-encoded resistance determinants and/or chromosomal determinants play a decisive role in heavy metal resistance in bacteria^3^. Anyway, no heavy metal-resistant plasmids are known to be present in the eukaryotes. Reportedly, there are also obvious differences between low and high Cu resistance in paths, which are determined only by several genes in yeasts *Saccharomyces cerevisiae*, *D*. *melanogaster*, and mammals^6^, respectively. Notably, even in the yeasts, some regulatory components in some machinery differ between the species such as *S*. *cerevisiae* and *S*. *pombe*^9^. However, detailed paths still need bridging.

Filamentous fungi are eukaryotes but unlike eukaryotic fungi yeasts, which are a collection of various unrelated fungi that are maybe classified into different taxa but all characterized by growth as filamentous hyphae cells and with a complex morphology (http://en.wikipedia.org/wiki/Fungus). Understanding of Cu resistance mechanisms is not only of significance in Cu utilization^10^ but also can gains insights into environmental and basic living processes of this group of fungi^11^. However, the Cu resistance-related paths and determinants in filamentous fungi remain unknown. In these regards, the related preliminary issues include differences in paths related to low and high Cu resistances, major potential substances against Cu, Cu effects on basal metabolism, correlation of accumulation of such osmolytes as proline with Cu resistance, real roles of superoxide dismutase (SOD) and peroxidase (POD) isoforms in Cu resistance, the networks for Cu compartmentalization, and Cu signaling pathways.

We previously reported *Penicillium janthinellum* strain GXCR, which was isolated from mining sludge, showing very high resistance to Cu^12^. In this study, we achieved the Cu resistance-decreasing mutants from the wild-type (WT) GXCR through mutagenesis by two different composite strategies of ethyl methane sulphonate—^60^Coγ irradiation(EC) and ultraviolet irradiation—^60^Coγ irradiation(UC), characterized differences of GXCR and the mutants in physiological and biochemical responses to Cu, and then conducted large-scale transcriptome sequencing to chart the mechanisms of filamentous fungi to Cu.

## Results

### Properties of the mutants

**Figure 1:**
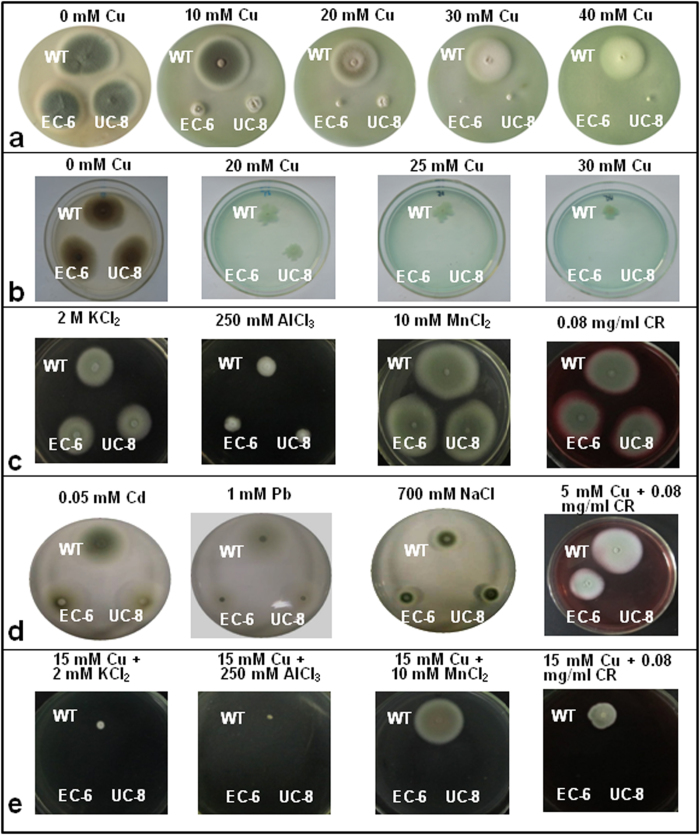
Phenotypes of *P*. *janthinellum* strain GXCR, and its mutants grown on PDA (***a***) TYA (***b***) and PDA (***c**, d,* and ***e***). For observation of phenotypes of the fungi, the mycelial discs were punched out with a 7-mm diameter sterilized cork borer from the colony margin of the 7-d-old colonies grown on PDA plate without external metals/antifungal substances, and then transferred to and grown for 7 d at 32 °C on PDA or TYA plate with or without external metals/antifungal substances. After that, the culture was photographed. The mutants, EC-6 and UC-8, were all characterized either by a smaller/tinier colony or by failing to form a colony on the media plate with external metals/antifungal substances, showing a decreased resistance relative to WT. CR, Congo red; EC, ethyl methane sulphonate—^60^Coγ irradiation; EC-6, the mutant resulted from EC mutagenesis; PDA, potato dextrose agar; TYA, TY agar; UC, ultraviolet irradiation—^60^Coγ irradiation; UC-8, the mutant resulted from UC mutagenesis; WT, wild-type.

EC-based mutagenesis resulted in a mutant, numbered EC-6, and UC-based mutagenesis brought about a mutant, numbered UC-8. These two mutants all presented Cu resistance significantly (P < 0.05) lower than WT strain GXCR, of which EC-6 was the lowest in Cu resistance (Fig. 1a). In addition to decreased Cu resistance, they did not differ from WT in growth rate and colony morphology when grown on potato dextrose agar (PDA) plate without external Cu (Fig. 1a).

When grown on organic nutrient-less TY^13^ agar (TYA) plate, EC-6 and UC-8 decreased much more in Cu resistance because they could not grow in the presence of 20 mM and 25 mM external Cu (Fig. 1b), respectively.

No remarkable differences between WT and the mutants were observed in resistance to K, Al, Mn, and Congo red (CR) (Fig. 1c), and Cd, Pb, and Na (Fig. 1d). EC-6 grew well under a combinative stress of Cu (5 mM) and CR (0.08 mg/mL) but UC-8 could not (Fig. 1d). However, both of them almost lost resistance to a combinative stress of Cu(15 mM) and K(2 mM), Cu(15 mM) and Al(250 mM), Cu(15 mM) and Mn(10 mM), or Cu (15 mM) and CR (0.08 mg/mL) (Fig. 1e).

**Figure 2:**
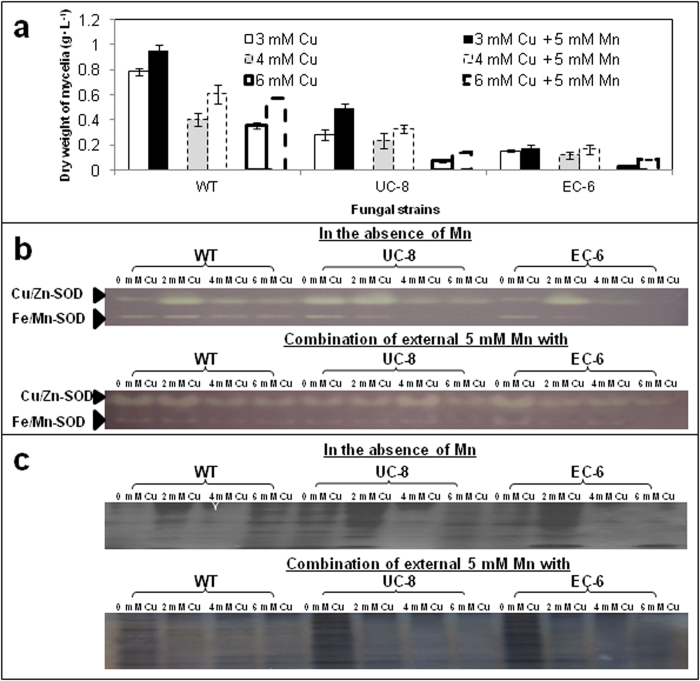
Dry weight (***a***) of, SOD (***b***) and POD (***c***) isoform activity inside the mycelia. For dry weight of mycelia, a 100-μL aliquot of conidial suspension (1 × 10^6^ condia/mL) was cultured for 48 h at 32 °C in LTY. For assay of activities of SOD and POD isoforms, a 100-μL aliquot of the conidial suspension of 1 × 10^6^ conidia/mL was added to 200-mL LTY with or without external heavy metals, cultured for 48 h at 32 °C in an incubator-shaker at 200 rpm, and then used for extraction of the crude enzyme solution. The crude enzyme solution was assayed by native non-denaturing polyacrylamide gel electrophoresis. The data of the dry weight of mycelia were the mean ± SD from three independent batches of experiments. The assay of isoform activity was repeated three times. EC, ethyl methane sulphonate—^60^Coγ irradiation; EC-6, the mutant resulted from EC mutagenesis; LTY, liquid TY; POD, peroxidase; SD, standard deviation; SOD, superoxide dismutase; UC, ultraviolet irradiation—^60^Coγ irradiation(UC); UC-8, the mutant resulted from UC mutagenesis; WT, wild-type.

**Mn effects on Cu resistance, and activities of SOD and POD isoforms**. It was presumed that Cu toxicity is likely alleviated by appropriate external Mn^14^. Consequently, when compared to growth in the presence of single Cu, production of mycelia was significantly (P < 0.05) increased for WT in the co-presence of 5 mM Mn and 3 (4 and 6) mM Cu, and for UC-8 only in the co-presence of Mn and 3 mM Cu (Fig. 2a). However, the production of EC-6 mycelia could not significantly (P < 0.05) alter in the co-presence of 5 mM Mn and Cu (Fig. 2a). These results suggest that external Mn can indeed detoxify the Cu toxicity, depending on the fungal strains.

In the presence of external Cu, Cu/Zn-SOD activity peaked in all the strains at 2 mM Cu. It was, however, undetectable in EC-6 at 6 mM Cu (Fig. 2b). No Fe/Mn-SOD activity was detectable in two mutants at 4 and 6 mM Cu (Fig. 2b).

Comparatively, in the co-presence of Mn and Cu, Cu/Zn-SOD activity was considerably enhanced in all the strains, and also appeared in EC-6 at 6 mM Cu (Fig. 2b). Fe/Mn-SOD activity was also visible in UC-8 at 4 and 6 mM Cu, but still not observed in EC-6 at 6 mM Cu (Fig. 2b).

Overall, changes in the total POD activity were very similar to changes in Cu/Zn-SOD activity in the presence of Cu, except the unexpected increase in WT and UC-8 at 6 mM Cu (Fig. 2c). The total activity either in WT or in the mutants was considerably repressed in the co-presence of Cu and Mn when compared to that under Cu-free control without external Mn (Fig. 2c). Interestingly, the POD activity was much higher in both UC-8 and EC-6 than in WT under Cu-free but Mn-containing control (Fig. 2c).

### Intracellular heavy metal accumulation

**Figure 3:**
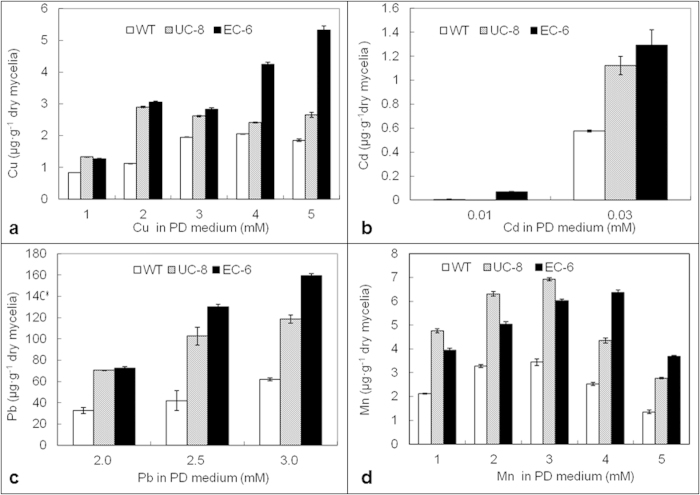
The content of Cu (***a***) Cd (***b***) Pb (***c***) and Mn (***d***) inside the mycelia. A 100-μL aliquot of conidial suspension (1 × 10^6^ condia/mL) was cultured for 48 h at 32 °C in LPD in an incubator-shaker at 200 rpm. The resulting mycelia were used for assay of heavy metal content by using flame AAS. The data were the mean ± SD from three independent batches of experiments. The mutants, EC-6 and UC-8, were characterized by higher intracellular heavy metal contents relative to WT. AAS, flame atomic absorption spectrometry; EC, ethyl methane sulphonate—^60^Coγ irradiation; EC-6, represents the mutant resulted from EC mutagenesis; LPD, liquid potato dextrose; SD, standard deviation; UC, ultraviolet irradiation—^60^Coγ irradiation; UC-8, the mutant resulted from UC mutagenesis; WT, wild-type.

Intracellular contents of Cu (Fig. 3a), Cd (Fig. 3b), Pb (Fig. 3c) and Mn (Fig. 3d) were significantly (P < 0.05) increased with increasing external concentrations of the corresponding heavy metals. Overall, the contents were much higher in two mutants than in WT. Notably, among the strains was that the EC-6 had the highest contents of Cu (Fig. 3a), Cd (Fig. 3b) and Pb (Fig. 3c).

The Mn content was significantly (P < 0.05) lower in EC-6 than in UC-8 if the external Mn was at and below 3 mM, but significantly (P < 0.05) higher in UC-8 than in EC-6 when the external Mn was at 4 and 5 mM (Fig. 3d), seemingly correlating with changes of two mutants in Fe/Mn-SOD isoform activity (Fig. 2b).

### **I**ntracellular reactive oxidative species (ROS)

**Figure 4:**
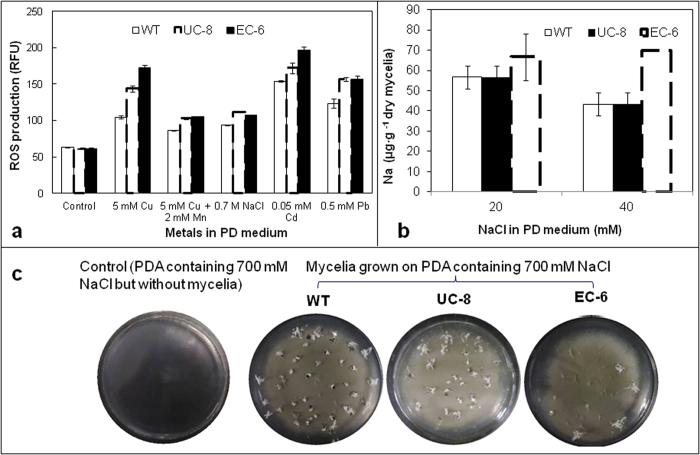
The ROS production (***a***) Na content inside the mycelia (***b***) and salting-out from the mycelia (***c***). Na content was assayed by flame AAS upon the mycelia cultured with a 100-μL aliquot of conidial suspension (1 × 10^6^ condia/mL) for 48 h at 32 °C in LPD in an incubator-shaker at 200 rpm. The data were the mean ± SD from three independent batches of experiments. The salting-out was conducted for 35 d at 32 °C on NaCl-containing PDA. The mutants, EC-6 and UC-8, were characterized by higher ROS production(***a***) higher Na content(***b***) and less Na-containing crystals on the surface of the colony (***c***) relative to WT in the presence of external metals. AAS, atomic absorption spectrometry; EC, ethyl methane sulphonate—^60^Coγ irradiation; EC-6, represents the mutant resulted from EC mutagenesis; LPD, liquid potato dextrose; PDA, potato dextrose agar; RFU, relative fluorescence unit; ROS, reactive oxidative species; SD, standard deviation; UC, ultraviolet irradiation—^60^Coγ irradiation; UC-8, the mutant resulted from UC mutagenesis; WT, wild-type.

Heavy metal stresses can lead to oxidative stress damages to the cells by triggering overproduction of some oxidative matters, especially ROS^15^,^16^. As a result, the WT and mutants showed no significant differences in intracellular ROS production under control without additional metals (Fig. 4a). However, both mutants showed a ROS production much higher than that in WT in the presence of external metals (Fig. 4a). Notably, ROS production either in WT or in mutants was much lower in co-presence of external Cu and Mn than in the presence of Cu (Fig. 4a), correlating with their growth in the co-presence of Cu and Mn (Fig. 2a).

### Na accumulation in and Na extrusion out of the mycelia

Reportedly, Na could be extruded out of cells through various channels/antiporters such as aquaporin-like channels in archea^17^ and P-type ATPases in yeast^18^. Consequently, Na content was the highest in EC-6 among the three strains in the presence of Na, implying that Na extrusion was severely impaired in EC-6. However, WT and UC-8 did not show a significant difference in Na content (Fig. 4b). We further tested Na extrusion from the mycelia on Na-containing PDA plate. As expected, Na extruded by the cells is much lower in EC-6 than in either of WT and UC-8 because very sparse white star-like crystals appeared on the colony surface of EC-6 (Fig. 4c), where the crystals were identified as Na-rich matters by silver nitrate precipitation reaction together with flame color method (data not shown).

### Aggregation of germinating conidia

**Figure 5:**
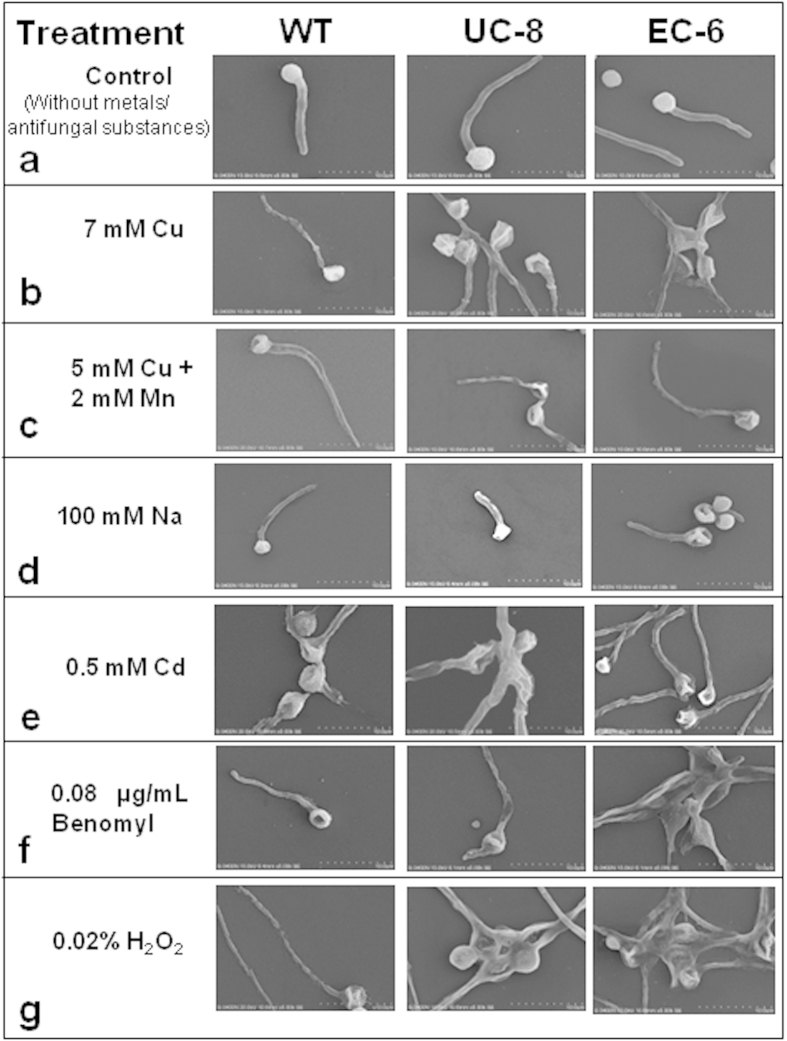
Behavior of the germinating conidia on PDA plate without metals/antifungal substances (***a***) and containing Cu (***b***) Cu + Mn (***c***) Na (*d*) Cd (***e***) Benomyl (***f***) and H_2_O_2_ (***g***) respectively. The observation of behavior of the germinating conidia was based on the cover-slip technique. In brief, a 10-uL aliquot of such conidial suspension was spread by using a 10-uL Pipette Tip on the surface of the microscopic cover-slip which were half-inserted in in tilted position with an angle of about 45° into PDA plate in Petri dish, and the culture was gown at 32 °C until the conidia germination and then observed by SEM. The photos were taken from the germinating conidia located at the interface between the covers-lip and the medium. The experiments were repeated three times. The mutants, EC-6 and UC-8, were all featured by aggregation of the more germinating conidia than WT in the presence of metals/antifungal substances. EC, ethyl methane sulphonate—^60^Coγ irradiation; EC-6, the mutant resulted from EC mutagenesis; PDA, potato dextrose agar; SEM, scanning electron microscope; UC, ultraviolet irradiation—^60^Coγ irradiation; UC-8, the mutant resulted from UC mutagenesis; WT, wild-type.

When exposed to heavy metals, the fungi have initial adaptive behaviors which reflect the tolerance development with increasing metal concentrations^19^. In this study, the germinating conidia of both WT and mutants showed no obvious aggregation in the absence of the external antifungal substances (Fig. 5a). In the presence of Cu, the remarkable aggregation was observed on the mutants rather than WT (Fig. 5b). However, the aggregation did not occur on three strains in the co-presence of Cu and Mn (Fig. 5c), indirectly supporting that external Mn can indeed detoxify the Cu toxicity (Fig. 2a). The aggregation was less under external Na (Fig. 5d), more obvious especially on two mutants under external Cd (Fig. 5e) and Benomyl (Fig. 5f), and the most significant especially on EC-6 under H_2_O_2_ (Fig. 5g).

### Proline content

**Figure 6:**
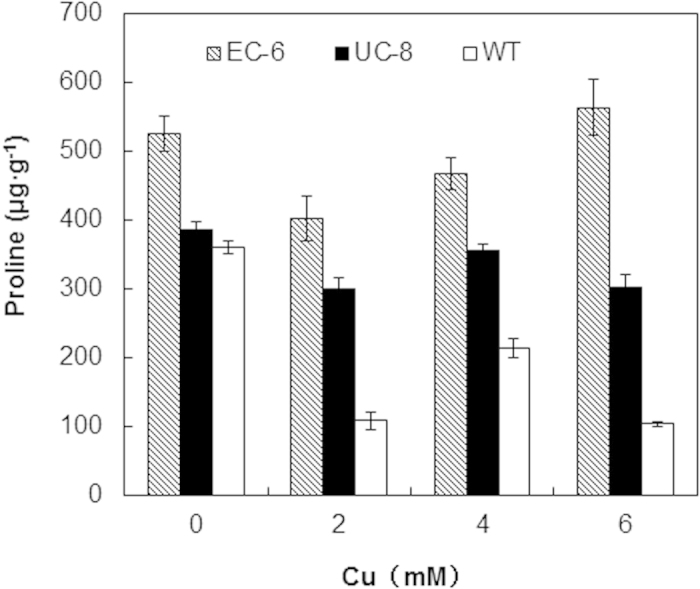
Proline content inside the mycelia. The proline content was estimated upon OD_520 nm_ value of the mycelia extract against the standard proline curve. The analysis approaches were detailed in Methods. The mutants, EC-6 and UC-8, were all highlighted by a proline content significantly (P < 0.05) higher than that of WD in the presence of external Cu. The data were the means ± SD from four independent batches of experiments. EC, ethyl methane sulphonate—^60^Coγ irradiation; EC-6, the mutant resulted from EC mutagenesis; OD, optical density; PDA, potato dextrose agar; SD, standard deviation; UC, ultraviolet irradiation—^60^Coγ irradiation; UC-8, the mutant resulted from UC mutagenesis; WT, wild-type.

Proline could not only act as a compatible osmolyte and a nonenzymatic antioxidant but also role in stabilizing protein structure^20^. Its accumulation has causal relationship with tolerance to various stresses^21^. The proline content in the mycelia was, on the whole, higher under Cu-free control than in the presence of external Cu. However, it obviously tended to decrease at 2 mM Cu, turned to significantly (P < 0.05) increase at 4 mM Cu, and decreased again 6 mM Cu in WT and UC-8 (Fig. 6). Uniquely, the proline content in EC-6 was significantly (P < 0.05) increased with increasing external Cu (Fig. 6).

### Transcriptome changes

**Figure 7:**
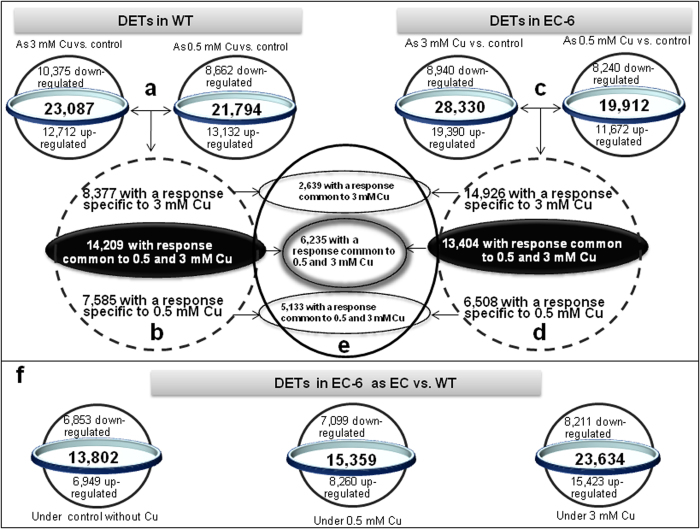
DETs in WT (***a*** and ***b***) DETs in the mutant EC-6 (***c*** and ***d***) DETs co-regulated in both WT and EC-6 (***e***) as well as DETs in EC-6 when compared as EC-6 vs. WT (***f***). The mycelia grown for 48 h at 32 °C in LTY with or without external Cu were used for transcriptome sequencing following Illumina sequencing technology. Definition of the DETs was described in detail in Methods. DETs, differentially expressed transcripts; EC, ethyl methane sulphonate—^60^Coγ irradiation; EC-6, the mutant resulted from EC mutagenesis. LTY, liquid TY; WT, wild-type.

In fungal cells, transcriptional regulatory mechanisms play a central role in response to the essential metals such as Cu^22^. Considering that EC-6 had the lowest Cu resistance (Figs. 1a and 1b), we sequenced a total of 6 transcriptomes from WT and EC-6 grown in liquid TY (LTY) under Cu-free control and in the presence of 0.5 and 3 mM Cu, respectively. Consequently, a total of 129 million reads with an average length of 93.60 bp were generated with a data amount of over 2G each transcriptome, of which the valid read ratio accounted for 85.51%. All valid reads from all the transcriptomes were collected together and then subjected to sequence splicing, consequently resulting in 47,407 transcripts of >93.60 bp in length. After removal of the repeats, 32,585 unigenes were obtained, of which the length of 4,424 unigens was over 2,000 bp. The sequences of all the valid transcripts have been released in GenBank nucleotide database (http://www.ncbi.nlm.nih.gov/nuccore/) under accession number from GBSP01000001 to GBSQ01013184 for sequences of >200 bp, and in GenBank SRA database under one accession number SRX749648 for all sequences of <200 bp (http://www.ncbi.nlm.nih.gov/sra/?term=SRP047270), respectively. The transcripts were mainly homologous to those from 10 species including *Penicillium*, *Aspergillus* and uncultured fungus, involving 100,263 reads. The detailed information on sequencing, annotation and pathways was presented in Supplementary Tables S1, S2, S3, S4 and S5. The presented here are differentially expressed transcripts (DETs) (Fig. 7). When compared as WT(Cu stress) vs. WT(control) and EC-6(Cu stress) vs. EC-6(control), respectively, the DETs were indicated in Figs. 7a–e). When compared as EC vs. WT, the DETs in EC-6 were shown by Fig. 7f.

Of the DETs, there were various categories of uniporters, symporters and/or antiporters, enzymatic and nonenzymatic antioxidants, signaling, and other stress-responsive proteins (Supplementary Tables S1, S2, S3, S4 and S5).

## Discussion

It was questionable whether multiple tolerance to different heavy metals and co-tolerance to combinations of several heavy metals are present in the living organisms^23^ although there was a view that many non-specific ‘open gates’ could bring in different metal ions even during metal excess^24^ because these ‘open gates’ were built up by some non-specific transporters that are responsible for tolerance to several metals^25^,^26^. In this study, all three fungal strains could show, to a certain extent, tolerance to the individual metals (Figs. 1a,c,d) but they were more sensitive to combinations of two metals (Fig. 1e). Interestingly, Cu resistance was also linked to resistance to CR (Fig. 1e), an inhibitor for wall biosynthesis of yeasts and some filamentous fungi^27^, suggesting that the cell wall is the first barrier of anti-Cu. Therefore, multiple resistance and co-tolerance to heavy metals indeed exist in filamentous fungi. Additionally, a reasonable speculation is that the integrity or thickness of the cells is an ignored factor for high resistance of the fungi to heavy metals.

Some evidence alluded that mycelial aggregation plays an active role in heavy metal resistance in some fungal species^28^,^29^. It was also found that some but not all of fungi showed conidial aggregation after the breaking of dormancy^30^, which likely involves unknown cell–cell interaction^29^. Our results showed that aggregation of the germinating conidia was more obvious in Cu resistance-decreasing mutants than in WT (Fig. 5). This seems to be reasonable because fast, effective, and early behavioral response is most important for microbes to survive the severe surroundings^31^. Taken together, initial aggregation of germinating conidia is an early important behavioral mechanism to resist Cu in fungi, with a feature that the strains with weaker Cu resistance have more aggregation of germinating conidia.

ROS detoxification depends partly on enzymatic antioxidants^32^, which are usually catalase, catalase/peroxidases (KatGs), PODs, and SODs^33^,^34^,^35^. SODs together with PODs form the first line of antioxidant defense against ROS^36^, of which SODs degrade O_2_^-1^ into H_2_O_2_ and various PODs then decompose H_2_O_2_ into H_2_O and O_2_^37^,^38^. In filamentous fungi, Fe/Mn-SOD is found exclusively in the mitochondria and Cu/Zn-SOD is predominant in the cytosol^39^. The higher Cu/Zn-SOD activity than Fe/Mn-SOD activity (Fig. 2b) was in agreement with a previous conclusion^39^. For EC-6, the lower activities of total SODs (Fig. 2b) and PODs (Fig. 2c) under higher Cu (4 and 6 mM) correlated well with lower resistance to higher Cu (Figs. 1a,b). In yeast *S*. *cerevisiae*, alleviation of growth inhibition under high Cu by external Mn was attributed to decrease in Cu accumulation^40^. Our results clearly indicated that the alleviation was also because the external Mn lowered ROS production (Fig. 4a) through increasing SOD activity especially at higher Cu (4 mM) (Fig. 2b). When compared as WT(Cu stress) vs. WT(control) and EC-6(Cu stress) vs. EC-6(control), respectively, a total of 52 and 56 enzymatic antioxidant DETs, including DETs of PODs and Fe/Mn-SOD, were found in WT and EC-6 under Cu, respectively. Of these DETs, nearly half (44-52%) was expressed in a down-regulation under Cu (Supplementary Tables S1 and S2). When compared as EC-6 vs. WT, about 35% of enzymatic antioxidant DETs showed a down-regulated expression (Supplementary Table S3), with POD DETs as the majority (at least 89%). Together with repressed POD activity under a combination of Cu and Mn (Fig. 2c), it can be therefore concluded that: high Cu can lead to failure in SOD-catalyzed degradation of O_2_^-1^, and impair the antioxidation catalyzed by Fe/Mn-SOD located in the mitochondria; and detoxification of ROS under high Cu depends on the non-enzymatic antioxidants rather than enzymatic antioxidants, agreeing with the previous viewpoint^15^.

The metal homeostasis depends largely on a balance between metal influx into and efflux from the cells, which have been reported in various types of species such as yeast^41^, bacteria^11^,^17^, crustaceans^8^ and plants^42^. Increase in intracellular heavy metal content with increasing external heavy metals (Fig. 3) implied that the metal uptake systems were still open even under high Cu. The likely reason for this is that the uptake systems usually employ many metal cotransporters/symtransporters, such as members belonging to Nramp family^43^. In EC-6, the decreased Na extrusion (Fig. 4c) correlated well with both Cu resistance level (Figs. 1a–c) and the higher ROS production (Fig. 4a). When compared as EC-6 vs. WT, a total of 44, 57 and 64 DETs related to metal-transporters were found in EC-6 under 0, 0.5 and 3 mM Cu (Supplementary Table S3), respectively. Of these DETs, the down-regulated DETs accounted at least for 82%, of which ATP7 DETs accounted at least for 56% (Supplementary Table S3). In yeast and mammals, ATP7 can function in Cu extrusion out of the cell by changing its subcellular localization^6^. Therefore, high Cu accumulation in EC-6 is likely due to failure in Cu extrusion (Fig. 3a), which resulted from disability of translocation of inner Cu. Taken together, Cu resistance of fungi is mainly determined by efficiently extruding Cu not by limiting Cu influx.

In yeast, Cu resistance is based on transporters Ctr1, Ctr3 and Fet4 located in the plasma membrane; Ccc2 located in the trans-Golgi network; Atx1, CCS and Sod1 located in cytosol; Sco1, COX and Cox17 in inner mitochondrial membrane; and Ctr2 located in vacuole membrane^4^. However, in mammals, SOD1 can be loaded by a copper–glutathione complex^7^. COX is an integral membrane mitochondrial enzyme and assembled by the inner mitochondrial membrane proteins Cox1, Cox2 Cox17, Cox11 and Sco1, of which Cox17 is responsible specifically for delivery of copper to Cox11 and Sco1^44^. For both Cox1 and Cox2, the catalytic subunits require Cu as a co-factor and are expressed from the mitochondrial genome^4^, and Sco1 is a nuclear gene-encoded protein found in yeast^45^. A total of 6 DETs encoding cytochrome c oxidase (CCO) assembly proteins, COX2, COX3, COX4, COX6A, COX6B and COX11, were expressed in an up-regulation in either of WT and EC-6 under Cu, of which COX2 was specific to low Cu (0.5 mM), and COX4, COX6A, COX6B and COX11 were specific to high Cu (3 mM) (Supplementary Tables S1 and S2). Curiously, no ScoI homolog and COX17 DETs were found in our sequencing repertoire. On the contrary, a number of DETs related to metal ion-binding proteins located in the innermembrane space of the mitochondria were found under Cu (Supplementary Tables S1 and S2). All these results indicate that there exists an unusual route of Cu delivery and/or distribution between multiple subunits of the mitochondrial CCO or between the innermembrane space and the innermembrane of the mitochondria in filamentous fungi. It should be indicate that among the DETs included several DETs of Fet3 and its partner Ftr1, of which Fet3 is a multicopper-ferroxidase essential for high-affinity iron uptake and it together with Ftr1 play a role in Cu extrusion out of cells in yeasts^4^. By these results, it could be extrapolated that such Cu delivery and/or distribution in the CCO can help buff high Cu stress on mitochondria, and therefore maintain the stability of the electron transport chain devices. Atx1 is a metallochaperone-like copper chaperone that sequesters and delivers Cu to Golgi^4^. When compared as WT(Cu stress) vs. WT(control) and EC-6(Cu stress) vs. EC-6(control), respectively, 12 (92.3%) of the Atx1 DETs were expressed in an up-regulation in WT under Cu (Supplementary Table S1). Oppositely, only one Atx1 DET showed down-regulated expression in EC-6 under high Cu (Supplementary Table S2). when compared as EC-6 vs. WT, 10 (76.9%) of the Atx1 DETs were down-regulated in EC-6 in a way specific to 0.5 mM Cu (Supplementary Table S3). All these results suggest that high expression of Atx1 is one of the mechanisms to handle high Cu.

Metal transport is partly dependent on active ATP^46^. When compared as EC-6 vs. WT, a total of 63, 65 and 92 DETs of ATP-dependent cation transporter were found in EC-6 under 0, 0.5 and 3 mM Cu (Supplementary Tables S3), respectively. Of these DETs, the down-regulated DETs accounted for 72.8-82.5%, with the DETs related to diverse V-ATPases and F-ATPases as the vast majority (Supplementary Table S3). V-ATPases function as proton pumps in diverse endomembrane organelles and plasma membranes^47^; The F-ATPases role in proton pumps but they are exclusively localized to the mitochondrial innermembrane and operate predominantly as an ATP-synthase^47^. By hydrolysis of ATP, the V-ATPases provide an electrochemical proton gradient to facilitate proton-coupled amino acid transport^48^. Taken together, the power resulting from ATP hydrolysis is the most important for Cu shuttle/transport associated with Cu resistance; Both F-ATPase-based ATP production and V-ATPases-based transmembrane transport constitute a power network responsible for Cu compartmentalization conferring Cu resistance.

Low Cu resistance is regulated by transcription factors(TFs) such as Mac1 in *S*. *cerevisiae*, dMTF-1 in *D*. *melanogaster*, and general TFs in mammals^6^. However, high Cu resistance depends on metallothionein(MT) systems regulated by such TFs as Ace1 in the yeast, dMTF-1 in *Drosophila,* and MTF-1 in mammals^6^. We only found six MT and MT-like DETs but did not search out homomolgs of Mac1, Ace1, dMTF-1, and MTF-1 from our sequencing repertoire (Supplementary Tables S1 and S2). However, numbers of DETs of general TFs were found in the Cu-stressed fungal strains (Supplementary Tables S1, S2, S3, S4 and S5). Therefore, as in mammals, resistance of filamentous fungi to low or high Cu is regulated largely by general TFs, somewhat differing from yeasts and *Drosophila*.

All protein-protein interactions are based on proline-dependent recognition^49^. Additionally, proline is an important precursor for production of the glutathione and its derivates such as glutamate that are all in a central role in detoxification of ROS and heavy metals^50^. When compared as EC-6 vs. WT, 20 (76.9%) of 26 DETs related to the proline utilization trans-activators were down-regulated in EC in a way specific to 3 mM Cu (Supplementary Table S5). Meanwhile, 37 (60.7%) of 61 DETs related to glutathione and its derivates showed an up-regulated expression in a way specific to 3 mM Cu, much higher than the related DETs specific to 0.5 mM Cu (Supplementary Table S5). When compared as WT(Cu stress) vs. WT(control) and EC-6(Cu stress) vs. EC-6(control), respectively, there were 51 to 83 DETs related to glutathione and glutamate, of which the up-regulated DETs accounted for about 75-79% in either of WT (Supplementary Table S1) and EC-6 (Supplementary Table S2) under high Cu. Interestingly, the proline content was significantly (P < 0.05) higher in EC-6 and UC-8 than in WT (Fig. 6). Anyway, lower proline content (Fig. 6) in the WT was not in contradiction with the previous conclusion that a higher proline level can maintain a higher level of the glutathione^50^. These results strongly suggest in filamentous fungi high Cu resistance is associated with efficient utilization of proline instead of with high accumulation of proline itself.

Some fungi such as yeast *S*. *cerevisiae* could not rely on MT synthesis as one of the Cu-resistance mechanisms under a combination of Cu and Cd^40^. Interestingly, only 2-3 of 6 MT-like DETs were found in either of WT and EC-6 to commonly respond to 0.5 and 3 mM Cu (Supplementary Tables S1 and S2), suggesting that MTs are not key players against Cu in filamentous fungi. Nonenzymatic antioxidants usually include betaines^51^; mannitol, melanin, and trehalose^52^; ascorbate, glutathione, carotenoids, tocopherols, and phenolics^53^; and amino acids including proline^20^. Additionally, the amino acids function in building blocks of the proteins^54^ and plant resistance to heavy metals^55^. As EC-6 vs. WT, there were a total of 15,064 DETs in EC-6 which had an expression exclusively specific to 3 mM Cu (Supplementary Table S5), of which 452 (3%) belonged to EDTs of nonenzymatic antioxidants, much higher than the number of the DETs specific to 0 or 0.5 mM Cu (Supplementary Table S5). Of these DETs, 316(~70%) were associated with metabolism and untilization of amino acids. Correspondingly, 77.2% of 145 DETs related to protein degradation were expressed in an up-regulation in EC-6 under 3 mM Cu, about 2-fold the corresponding DETs under 0 or 0.5 mM Cu (Supplementary Table S2), strongly suggesting that high Cu causes degradation of the proteins into amino acids. Taken together, amino acids resulting from protein degradation are a major category of nonenzymatic antioxidants in high Cu resistance.

The key basal metabolic pathways depend on the activated acyl groups such as Acyl-CoA^56^. One of routes regulating the cellular CoA:acyl-CoA ratio is through metabolism of carnitine, glycine betaine and choline^57^. Glycine betaine is produced by the betaine aldehyde dehydrogenases (betA)^58^ and through oxidation of choline by choline dehydrogenase(CHD) located in the inner membrane of mitochondria^59^. Carnitine is also produced by betA and buffers free CoA and Acyl-CoA pool through activated Acyl-CoA generated by carnitine acyltransferases(CA)^58^. CA has been found in mammalian mitochondria, peroxisomes and endoplasmic reticulum (ER)^56^. When compared as EC-6 vs. WT, a total of 67, 97 and 205 Acyl/CoA-related DETs were found in EC-6 under 0, 0.5 and 3 mM Cu, respectively. Of these DETs, DETs down-regulated by 3 mM Cu accounted for about 78%, of which DETs of CA accounted for 62.7% that were positioned on the peoxisomes pathways (Supplementary Table S3). When compared as WT(Cu stress) vs. WT(control) and EC-6(Cu stress) vs. EC-6(control), respectively, there were 199 and 245 Acyl/CoA-related DETs in WT under 0.5 and 3 mM Cu (Supplementary Table S1), respectively. Meantime, there were 144 and 191 Acyl/CoA-related DETs in EC-6 under 0.5 and 3 mM Cu (Supplementary Table S2), respectively. The up-regulated DETs in both strains accounted for at least 54.5%. Not all filamentous fungi harbor the peroxisomes^39^,^60^, and not all bacteria can convert choline to betaine^51^. Taken together, higher Cu can lead to imbalance of a buffering free CoA and Acyl-CoA pool in the peroxisome, and *P. janthinellum* can likely produce glycine betaine through the CHD-catalyzed conversion of choline.

In this study, many signaling pathways were found to respond to Cu stress, here we focus on the Ras system, the two-component system (TCS) and the osomolarity TCS (OTCS) (Supplementary Tables S1, S2, S3, S4 and S5). The Ras superfamily includes five major subfamilies: Ras, Rho, Arf/Sar, Ran and Rab, some members of which are membrane proteins and respond to the diverse extracellular stimuli through functions as signaling nodes^61^. The Ras system also cross-links with the signal recognition particle receptor (SRPR), which ensures that nascent secretory proteins (NSPs) are destined for the eukaryotic ER by interaction with cargo proteins^62^. The signaling can occur in a front–back communication inside Arfs/Sar proteins^63^ which have been found in yeasts but not in filamentous fungi through analysis of the available complete genome sequences^61^. As WT (Cu stress) vs. WT(control) and EC-6 (Cu stress) vs. EC-6(control), respectively, a total of 127 and 145 Ras DETs were found in WT under 0.5 and 3 mM Cu, respectively. Of these DETs, the majority (91-96%) were expressed in an up-regulation (Supplementary Table S1). A total of 136 and 152 Ras DETs were found in EC-6 under 0.5 and 3 mM Cu (Supplementary Table S2), respectively, of which up-regulated DETs accounted for about 88-94%. Correspondingly, 17 (85%) of 20 SRPR DETs were found in either of WT and EC-6 (Supplementary Tables S1 and S2). As EC-6 vs. WT, a total of 120 Ras DETs were found in EC-6 under 3 mM Cu, at least 6.6-fold Ras DETs regulated under control or by 0.5 mM Cu (Supplementary Table S3). Of the DETs regulated by 3 mM Cu, 94% were down-regulated. Meanwhile, a total of 7 SRPR DETs were all up-regulated in a way specific to 3 mM Cu (Supplementary Table S5), about 7-fold SRPR DETs regulated under control or by 0.5 mM Cu. These results together clearly indicated that up-regulated expression of Ras members is necessary to Cu resistance in filamentous fungi, and more NSPs could be targeted to ER under high Cu. TCSs participate in bacterial resistance to heavy metals^3^,^64^. So far, two types of TCSs with an element of histidine kinase or receiver domains have been reported in fungi^65^, but little is known about their association with heavy metal resistance of fungi. Sequencing uncovered 6 TCSs which were the barA, cph1, K11527, pleC1, rcsC, and torS (Supplementary Tables S1 and S2). As WT(Cu stress) vs. WT(control) and EC-6(Cu stress) vs. EC-6(control), respectively, the mainly responsive TCS was torS in either of WT and EC-6 (Supplementary Tables S1 and S2) because its DETs were all expressed in an up-regulation under 0.5 and 3 mM Cu. As EC-6 vs. WT, 64 (94%) of k11527 DETs was expressed in an up-regulation in EC-6 in a way specific to 3 mM Cu (Supplementary Table S5). Therefore, k11527 functions in preference to high Cu resistance. The discovered OTCS were SKN7, SLN1, SSK1, and YPD1. As WT(Cu stress) vs. WT(control) and EC-6(Cu stress) vs. EC-6(control), respectively, a total of about 6 OTCS DETs were found in either of WT and EC-6 under Cu (Supplementary Tables S1 and S2). Interestingly, there was a cross-talk among different signaling pathways (Supplementary Tables S1, S2, S3, S4 and S5).

In human, one of the main transporter groups is the solute carrier (SLC) superfamily composed of 55 subfamilies^66^. This superfamily can pump various metal ions out of cells or into organelles, therefore functioning in cell homeostasis^46^. As WT(Cu stress) vs. WT(control) and EC-6(Cu stress) vs. EC-6(control), respectively, a total of 70 and 110 SLC DETs were in WT under 0.5 and 3 mM Cu, respectively, of which the up-regulated DETs accounted for 70% at 0.5 mM Cu and 69% at 3 mM Cu (Supplementary Table S1). Similarly, 95 and 111 SLC DETs were in EC-6 under 0.5 and 3 mM Cu, respectively, of which the up-regulated DETs accounted for 58% at 0.5 mM Cu and 73.9% at 3 mM Cu (Supplementary Table S2). These DETs from a total of 37 SLC subfamilies, which mainly involved SLC subfamily 30; SLC subfamily 25 (mitochondrial phosphate transporter); SLC subfamily 39; and SLC subfamily 25 (mitochondrial carnitine/acylcarnitine transporter). As EC-6 vs. WT, a total of 46, 42 and 60 SLC DETs were in EC-6 under 0, 0.5 and 3 mM Cu (Supplementary Table S3), respectively. In these DETs, the down-regulated DETs accounted for 57.4%. Another concern was the transporters of major facilitator superfamilies (MFS) because they are the uniporters, symporters and/or antiporters^67^. As WT(Cu stress) vs. WT(control) and EC-6(Cu stress) vs. EC-6 (control), respectively, a total of 231 and 256 MFS DETs were in WT under 0.5 and 3 mM Cu (Supplementary Table S1), respectively. Meanwhile, a total of 187 and 288 MFS DETs were in EC-6 under 0.5 and 3 mM Cu (Supplementary Tables S2), respectively. As EC-6 vs. WT, a total of 92, 87 and 246 MFS DETs were found in EC-6 under 0, 0.5 and 3 mM Cu (Supplementary Table S3), respectively. Taken together, SLC and MFS are crucial in the solute compartmentalization transport related to Cu resistance.

The pre-mRNAs from over 60% of intron-containing genes undergo an alternative splicing (AS)^68^, highlighted by a vast repertoire of mRNA isoforms. The AS process involves the nuclear calmodulin (CaM)^69^ and can occur in large ribonucleoprotein machinery spliceosome^70^. In this study, we found that most of the genes have multiple mRNA isoforms under Cu stress, which more or less showed obvious differences in expression pattern under Cu stress. The evidence for AS occurring under Cu stress was that there were many DETs of CaM, pre-mRNA-splicing proteins (PMSs) and rRNA-processing proteins positioning on the spliceosome pathway (no. ko03040), splicing factors (SFs) on the spliceosome pathway, and tRNA modification GTPase (Supplementary Tables S1, S2, and S3). As WT(Cu stress) vs. WT(control) and EC-6(Cu stress) vs. EC-6(control), respectively, a total of 42 and 47 AS-related DETs were in WT under 0.5 and 3 mM Cu (Supplementary Table S1), respectively. A total of 60 and 65 AS-related DETs were in EC-6 under 0.5 and 3 mM Cu (Supplementary Table S2), respectively. Of these DETs, the major groups were PMSs and SFs, accounting for at least 47% of the DETs in either of WT and EC-6 under 3 mM Cu (Supplementary Tables S1 and S2). Interestingly, more than 75% of SF DETs in either of WT and EC-6 under Cu were down-regulated in expression, by contrast, more than 73% of the PMS DETs in either of WT and EC-6 under 3 mM Cu were expressed in an up-regulation (Supplementary Tables S1 and S2). Notably, the number of SF or PMS DETs in either of WT and EC-6 under 3 mM Cu was at least 1.4-fold that under 0.5 mM Cu. As EC-6 vs. WT, the number of SF or PMS DETs multiplied with increasing external Cu, and the number of the up-regulated DETs accounted for at least 60% under 3 mM Cu (Supplementary Tables S1 and S2). Taken together, active AS is associated with Cu resistance, which depends mainly on PMSs rather than SFs positioning on the spliceosome pathway.

**Figure 8:**
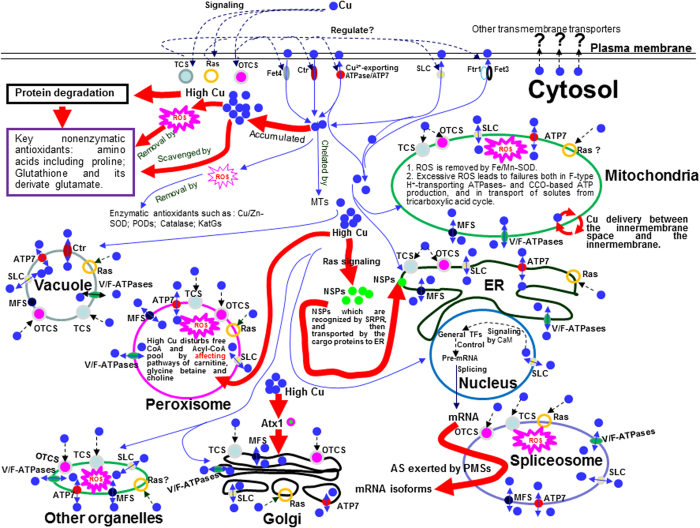
The suggested model on key paths and determinants of resistance of *P. janthinellum* strain GXCR to Cu. Indicated here are the key, but not all, paths and determinants for Cu delivery and signaling for tolerance to Cu, which were concluded by this study. The high Cu resistance follows the paths: (1) protein degradation, which produces nonenzymatic antioxidant of amino acids; (2) high expression of Atx1; (3) Cu delivery and distribution between multiple subunits of the mitochondrial CCO and/or between the innermembrane space and the innermembrane; (4) transport of SRPR-recognized NSPs to ER by the cargo proteins under Ras signaling; (5) pathways of carnitine, glycine betaine and choline that buffer free CoA and Acyl-CoA pool in the peroxisome; and (6) more active AS exerted mainly by PMSs rather than SFs positioned on the spliceosome pathway. The potential subcellular localization of the involved components were assumed by referring to the literature^4^,^6^,^7^,^15^,^32^,^33^,^34^,^35^,^37^,^38^,^39^,^44^,^45^,^46^,^47^,^56^,^61^,^62^,^64^,^65^,^66^,^67^,^69^,^70^. All the components are given an icon. The blue solid circles indicate Cu ion. The dashed black arrows represent the potential Cu signaling routes or some unknown Cu-extruding channels. The blue solid single arrows represent potential routes and destination of Cu delivery. The blue solid double arrows indicate Cu transport in and out of the organelles. The black solid arrow indicate the mRNA transport out of the nucleus. The thick solid red arrows indicate the metabolic events and/or the paths under high Cu. The explosive icons indicate the excessive ROS generated under high Cu. AS, alternative splicing; CaM, calmodulin; CCO, cytochrome c oxidase; Ctr, copper transporter; ER, endoplasmic reticulum; Fet3, Fe transport multicopper oxidase; Fet4, low affinity Cu/Fe transporter; Ftr1, high-affinity Fe transporter; KatGs, catalase/peroxidases; MFS, major facilitator; MTs, metallothioneins; NSPs, nascent secretory proteins; OTCS: osomolarity TCS; PMSs, pre-mRNA-splicing proteins; POD, peroxidase; Ras, Ras superfamily; ROS, reactive oxidative species; SFs, splicing factors; SLC, solute carrier; SOD, superoxide dismutase; SRPR, signal recognition particle receptor; TCS, two-component system; TFs, transcription factors; V/F-ATPases, V/F-type H^+^-transporting ATPases.

In summary, Cu resistance of filamentous fungi involves behavioral and molecular responses to Cu. The initial aggregation of germinating conidia is the first behavioral response to external Cu, weaker Cu resistance, more aggregation of germinating conidia. After entering the cells, Cu makes an impact on the basal metabolism through affecting metabolic pathways that buffer free CoA and Acyl-CoA pool in the peroxisome. The Cu resistance mechanisms involve Cu extrusion out of the cells mainly by some transporters, transporter-like proteins and/or solute carriers such as ATP7, SCLs, Fet3 and Ftr1 located in the plasma membrane; removal of intracellular ROS by nonenzymatic antioxidants such as amino acids and glutamate and by enzymatic antioxidants such as SODs, PODs, catalase and KatGs; Cu chelation/sequestration by MT and/or some organic matters such as amino acids; Cu compartmentalization through cargo proteins, MFS, NSPs, SLCs, SRPRs, and V/F-ATPases. These processes need a lot of ATP-based energy especially under high Cu, and involve various signaling pathways such as systems of Ras, TCSs and OTCSs. At the transcriptional level, the mechanisms are regulated largely by general TFs, somewhat differing from yeasts and *Drosophila*. It should be noted that there are no distinct differences between low and high Cu resistance in paths because there are an overlap and/or a cross-talk among many pathways responsive to low and high Cu partly through signaling pathways at the transcriptional level. However, there are indeed some backbone paths that play roles mainly in high Cu resistance, which follow (1) protein degradation, which produces nonenzymatic antioxidant of amino acids; (2) high expression of Atx1; (3) complicated Cu delivery and/or distribution between multiple subunits of the mitochondrial CCO and/or between the innermembrane space and the innermembrane of the mitochondrial; (4) transport of SRPR-recognized NSPs to ER by the cargo proteins under Ras signaling; (5) pathways of carnitine, glycine betaine and choline that buffer free CoA and Acyl-CoA pool in the peroxisome; and (6) more active AS exerted mainly by PMSs rather than SFs positioned on the spliceosome pathway. Taken all results together, a model on key paths and determinants of Cu tolerance of filamentous fungi was proposed in Fig. 8.

## Methods

### Fungal strains and growth conditions

The fungal strains used were *P*. *janthinellum* strain GXCR^12^, and its Cu resistance-decreasing mutants which were constructed as below methods in this study. The media used were conventional PDA; TYA^13^ that was composed of 10 g Bacto-tryptone, 5 g yeast extract, 5 g NaCI, 2 g sodium gluconate and 20% agar in 1,000 mL water; liquid potato dextrose (LPD) without agar; and liquid TY (LTY) without agar^13^.

The fungi were grown at 28 °C or 32 °C on solid medium plates, in liquid media with or without external metal salts, depending on the experiments.

### Composite mutagenesis

Two composite mutagenesis strategies, EC and UV, were used, respectively.

For EC mutagenesis, the fungal conidia were suspended and incubated for 18 h at 30 °C in 0.05% Tween-80 solution, and then mixed as 4:1 with the buffer solution composed of 1 mL of 0.5 M EMS and 20 mL phosphate buffer at pH 7.0. The mix was incubated for 140 min at 28 °C in an incubator-shaker at 100 rpm, and then centrifuged for 5 min at 9,000 rpm to collect the germinating conidia. The collected conidia were carefully washed three times with 5% Na_2_S_2_O_3_ and re-suspended in sterile ultrapure water, setting up the conidial suspension of 1 × 10^3^ conidia/mL. A 100-μL aliquot of the conidia suspension was spread on PDA plate and grown at 32 °C until the mycelia formed and produced the mature conidia. The resulting conidia were collected and suspended in 0.05% Tween-80 solution to generate the conidial suspension of 1 × 10^6^ conidia/mL. The conidial suspension was then irradiated by ^60^Coγ-ray of 0.5 Kgy at a dose rate of 20 GV/min. A 0.1-mL aliquot of the irradiated conidial suspension was spread on PDA plate without added CuSO_4_ and then grown for 72 h at 32 °C until the colonies formed. The resulting colonies were transferred as replica plating method to PDA plate with 20 mM CuSO_4_ to screen the mutants with decreased Cu resistance.

For UC mutagenesis, the conidia was spread on PDA plate, exposed for 75 s to UV light generated by a 15 W low-pressure mercury vapour lamp, and then cultured at 32 °C in dark until the mycelia formed and produced the mature conidia. The formed conidia were collected and suspended in 0.05% Tween-80 solution, establishing the conidial suspension of 1 × 10^6^ conidia/mL. A 2-mL aliquot of the conidial suspension was pipeted into the Eppendorf tuber, and then irradiated by ^60^Coγ-ray as the mentioned above. The screening of the mutants with decreased Cu resistance was conducted following the above-mentioned procedures.

Parallel controls were set up with untreated conidial preparations from WT.

### Preparation of crude enzyme solution

A 100-μL aliquot of the conidial suspension of 1 × 10^6^ conidia/mL was added to 200-mL LTY with or without external heavy metals, and cultured for 48 h at 32 °C in an incubator-shaker at 200 rpm. The resulting culture was collected through filtration with a double-layer of sterile medical gauze, and then washed twice with the sterile ultrapure water. The washed culture was placed for certain time on sterile filter paper to remove the remaining water. A 0.2-g aliquot of the culture was homogenized in 2 mL pre-cooled 4 × buffer (18.2% Tris buffer at pH 8.8) on ice. The homogenate was further treated for 10 min as procedures of a 10-s ultrasonic wave treatment followed by a 10-s interval under the rated power of 38% on ice. The homogenate was then centrifuged for 5 min at 4 °C at 8,603 × *g*. The resulting supernatant was stored at −80 °C as crude enzyme solution.

### Gel assay of activities of SOD and POD isoforms

The SOD isoform activity was assayed with the crude enzyme solution by native non-denaturing polyacrylamide gel electrophoresis. In brief, separating gels (10%) were prepared with 2.5 mL of 4 ×buffer composed of 18.2% Tris buffer at pH 8.8, 2.5 mL of 30% acrylamide, 15 μL of tetramethylethylenediamine (TEMED), 41.6 μL of 10% ammonium persulfate (AP), and 5 mL distilled water. Stacking gels (5%) were composed of 1.25 mL of 16% Tris buffer at pH 6.8, 625 μL of 30% acrylamide, 15 μL of TEMED, 30 μL of 10% AP, and 3.125 mL distilled water. A 20-μL aliquot of crude enzyme solutions was loaded on the gel for electrophoresis. The electrophoretic buffer solution was composed of 2.88% glycine and 0.6% Tris at pH 8.8. The electrophoresis was performed for 4 h at 4 °C at 120 V for separating gels and for 30 min at 4 °C at 100 V for stacking gels, respectively. After electrophoresis, the gel was fully washed twice with the distilled water, stained by using nitroblue tetrazolium, decolored and then fixed with 6% acetic acid at pH 4.7 to examine SOD isoforms. To examine POD isoforms, the gel was stained by using ascorbic acid acetate benzidine. The stained gels were photographed by using a Model GS-800^TM^ Calibrated Imaging Densitometer.

### Assay of intracellular metals

The intracellular metals were assayed through the flame atomic absorption spectrometry (AAS) with the ZEEnit700P atomic absorption spectrometer (Germany) equipped with the microwave digestion furnace as conventional procedures.

### Assay of ROS production

A 50-μL aliquot of conidial suspension of 1×10^6^ condia/mL was added to 100 mL LPD with or without external Cu, and cultured for 48 h at 32 °C in an incubator-shaker at 200 rpm. Then, an about 0.05-g aliquot of the resulting mycelia was transferred into the Ependoff tuber followed by adding 1-mL pre-cooled Reagent A solution and then gently mixed. The mix was centrifuged for 10 min at 3,000 rpm. After the supernatant was discarded, the sediment was suspended in a 1-mL aliquot of Genmed staining solution, and then placed for 20 min in 28 °C water bath in dark. Such mix was then centrifuged for 10 min at 1,000 × *g*, and the resulting supernatant was discarded. Following that, the sediment was suspended in a 500-μL aliquot of pre-cooled Reagent A solution. A 500-μL aliquot of the suspension was transferred into the 1-mL colorimetric Cuvette followed by adding a 500-μL aliquot of Reagent D solution, and then measured for fluorescence with a PerkinElmer LS55 Fluorescence spectrometer (US) at 490 nm as excitation wavelength and at 530 nm as emitting wavelength. The amount of ROS was expressed as relative fluorescence units (RFU). The Reagents A and D, and Genmed staining solution were from fungi/yeast reactive oxidative species high-quality fluorescence assay kit (Genmed Scientifics Inc. USA).

### Observation of aggregation of germinating conidia

The aggregation of germinating conidia was studied by using the cover-slip technique. A 1-mL aliquot of conidial suspension of 1 × 10^6^ conidia/mL was transferred to 2 mL of 0.05% tween-80, and then mixed by 1-min vortex. A 10-uL aliquot of such conidial suspension was spread by using a 10-uL Pipette Tip on the surface of the microscopic cover-slip which was half-inserted in tilted position with an angle of about 45° into PDA plate in Petri dish. The spreading ensured that the conidial suspension just contacted with the interface between the coverslip and the medium. After spreading, the Petri dish was covered with the lid and then placed at 32 °C until the conidia germination. The cover-slip loaded by germinating conidia was fixed as requirements by scanning electron microscope (SEM) and then observed by SEM (HITACHI S-3400). The observation focused on the germinating conidia located at the interface between the coverslip and the medium.

### Quantitation of proline

A 100-μL aliquot of conidial suspension of 1 × 10^6^ conidia/mL was inoculated in 200-mL LTY and cultured for 48 h at 32 °C in an incubator-shaker at 200 rpm. The resulting fungal culture was oven-dried, and then homogenized for 2 min in pre-cooled 3% sulfosalicylic acid on ice by using an ultrasonic homogenizer. The homogenate was centrifuged for 10 min at 4 °C at 6,000 × *g*. The supernatant was added by 200 μL glacial acetic acid and 200 μL ninhydrin, heated for 15 min in boiling water, and then immediately cooled to 0 °C on ice. The cooled solution was mixed with 500 μL toluene, and placed at room temperature in dark until it fully stratified. After the solution stratified, optical density (OD) of the supernatant was recorded at 520 nm. The proline content was therefore estimated against the standard curve prepared with the standard proline sample (Sigma) at OD_520 nm_ following the formula: Proline (μg·g^-1^ mycelia) = (C×Vt)/(W×Vs), where C indicated praline (μg) against the standard curve, Vt represented the total mycelial extract (mL), W represented the mycelia (g), and Vs represented the analyzed extract volume (mL).

### cDNA library preparation

The conidia were inoculated in 200 mL LTY medium with 0, 0.5 and 3 mM Cu, respectively, and cultured for 3 d at 32 °C in an incubator-shaker at 200 rpm. The mycelial cultures resulting from different conditions were separately used for extraction of the total RNA by using the TruSeq Stranded Total RNA LT-(with Ribo-Zero^TM^ Gold)-Set B according to the manufacturer’s protocol. The quality and integrity of the RNA sample were controlled by Bioanalyzer 2100 (Agilent, Germany) and by running agarose gel, respectively. A 5-μg aliquot of mRNA was captured from each kind of total RNA sample by Dynabeads Oligo(dT) (Life technologies, USA), sheared to fragments of ~200 bp, and then reverse-transcribed into cDNAs by using the SuperScriptIII cDNA Synthesis Kit (Life technologies, USA). The cDNAs were end-repaired by ligation of an A base followed by ligating to Illumina sequencing Truseq V2 RNA Adapter which was from the TruSeq RNA LT V2 Sample Prep Kit (Illumina, Sandiego). After the end modification, the cDNAs were amplified by 12 thermal cycles of PCR and then used for construction of the cDNA library by using the TruSeq RNA LT V2 Sample Prep Kit (Illumina, Sandiego).

### Transcriptome sequencing

Transcriptome sequencing was based on the cDNA libraries. The libraries were qualified by Qubit 1.0 (Life technologies, USA) and Bioanalyzer 2100 (Agilent, Germany) before sequencing. Sequencing was performed on the Illumina Hiseq 2000 sequencer by using the TruSeq SBS v3-HS kits as 2 × 100 bp paired-end strategy. The quality of the reads was controlled as the criteria of 20 (error rate = 1%) for quality threshold, 5 bp for window size, and 35 bp for length threshold. The N-containing sequences were removed from the reads following the standard of length threshold of 35 bp. The resulting valid reads from all the sequencing samples were collected, forming a valid read collection. A total of 500,000 reads which were randomly chosen from the collection were subjected to the initial homology analysis as the cut-off values of 1E < 10 and the 80% coverage against public nucleotide databases to examine contamination of and remove the contaminated reads. Subsequently, the valid reads underwent *de novo* splicing as the paired-end method by using the Trinity software (trinityrnaseq_r2011-11-26) under the default parameters of the software. The unigene was represented by the longest transcript under each locus.

### Functional annotation, clustering and pathway mapping of the unigenes

The unigens were functionally annotated as the cut-off values of both >30% homology and 1E≤−5 against public databases of nr, SWISS-PROT, TrEMBL, Cdd, pfam, and clusters of orthologous groups for eukaryotic complete genomes (KOG), and then functionally clustered through KOG approaches.

The mapping of the unigenes onto the pathways was based on the database resource of the kyoto encyclopedia of genes and genomes (KEGG) through KEGG Automatic Annotation Server (http//www.genome.jptools/kass/).

### Definition of DETs

The DETs were defined according to fold change calculated though log2 (A vs. B), where A and B represented the number of the samples A and B, respectively. If the log_2_ was >1 or <1, the gene was defined as the up-regulated expression or down-regulated expression, respectively.

### Statistical analysis

Statistical analyses were performed with SPSS. Differences between two variables in the mean were evaluated by the Student’s *t*-test and the Mann-Whitney U test. A *p* value of <0.05 was considered statistically significant.

## Additional Information

**How to cite this article**: Xu, J. *et al.* Paths and determinants for *Penicillium janthinellum* to resist low and high copper. *Sci. Rep.* **5**, 10590; doi: 10.1038/srep10590 (2015).

## Supplementary Material

Supplementary Table S1

Supplementary Table S2

Supplementary Table S3

Supplementary Table S4

Supplementary Table S5
